# Exploring the Molecular Dynamics of a Lipid-A Vesicle at the Atom Level: Morphology and Permeation Mechanism

**DOI:** 10.1021/acs.jpcb.3c02848

**Published:** 2023-07-19

**Authors:** Denys E. S. Santos, Antonio De Nicola, Vinicius F. dos Santos, Giuseppe Milano, Thereza A. Soares

**Affiliations:** † Departmento de Química Fundamental, Universidade Federal de Pernambuco, Recife 50740-560, Brazil; ‡ Scuola Superiore Meridionale, Largo S. Marcellino 10, Napoli 80138, Italy; § Graduate School of Organic Materials Science, Yamagata University, Yonezawa 992-8510, Yamagata, Japan; ∥ Departamento de Química, Faculdade de Filosofia, Ciências e Letras de Ribeirão Preto, Universidade de São Paulo, Ribeirão Preto 14040-901, Brazil; ⊥ Department of Chemical, Materials and Production Engineering, University of Naples Federico II, Piazzale Tecchio 80, Napoli 80125, Italy; # Hylleraas Centre for Quantum Molecular Sciences, University of Oslo, Oslo 0315, Norway

## Abstract

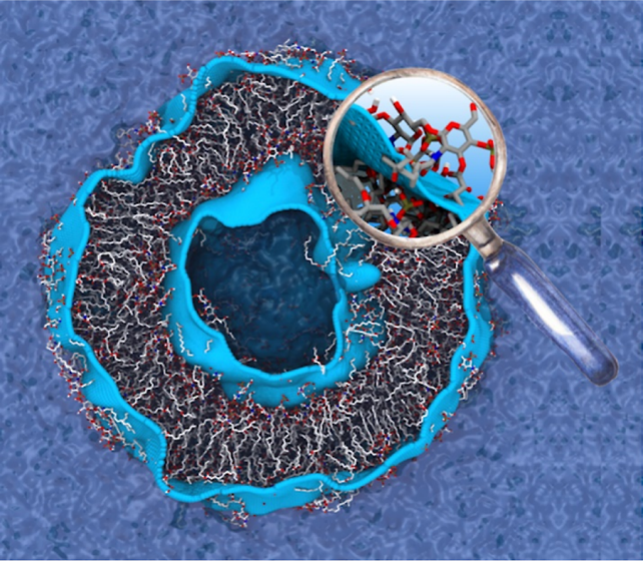

Lipid-A was previously shown to spontaneously aggregate into a vesicle *via* the hybrid particle field approach. We assess the validity of the proposed vesiculation mechanism by simulating the resulting lipid-A vesicle at the atom level. The spatial confinement imposed by the vesicle geometry on the conformation and packing of lipid-A induces significant heterogeneity of physical properties in the inner and outer leaflets. It also induces tighter molecular packing and lower acyl chain order compared to the lamellar arrangement. Around 5% of water molecules passively permeates the vesicle membrane inward and outward. The permeation is facilitated by interactions with water molecules that are transported across the membrane by a network of electrostatic interactions with the hydrogen bond donors/acceptors in the *N*-acetylglucosamine ring and upper region of the acyl chains of lipid-A. The permeation process takes place at low rates but still at higher frequencies than observed for the lamellar arrangement of lipid-A. These findings not only substantiate the proposed lipid-A vesiculation mechanism but also reveal the complex structural dynamics of an important nonlamellar arrangement of lipid-A.

## Introduction

The uniqueness of the outer membrane (OM) architecture in Gram-negative bacteria resides on the asymmetrical distribution of complex lipopolysaccharides. The OM inner leaflet is composed of phospholipids whereas the outer leaflet is composed of negatively charged lipopolysaccharides (LPSs) stabilized by numerous divalent cations. LPSs are highly specialized molecules found exclusively in the OM external leaflet where it provides a formidable barrier against xenobiotics and plays a critical role in host-pathogen virulence. Under environmental stimuli, Gram-negative bacteria activate stress response pathways to catalyze chemical modifications of the LPS structure. Such on-the-fly modifications constitute an efficient modulation mechanism of the OM physicochemical properties, enabling the organism to efficiently evade the host immune defense.

The lipid-A moiety of the LPS molecule is responsible for the toxic and immunostimulatory activity at *fmol* amounts.^1^ Lipid-A moieties are microbe-associated molecular patterns (MAMPs) and immune ligands for eukaryotic pattern recognition receptors (PRRs) which control inflammation, host immunity, and cell death in response to interactions with Gram-negative bacteria.^2^ In the blood, the LPS-binding protein (LBP) recognizes and binds the lipid-A moiety, mediating the extraction and disassembling of the aggregates.^3^ As the innate immune system defends against pathogens by detecting MAMPs using PRRs, the immune response is critically dependent on the lipid-A chemical structure.^4^ The reduction of the number of acyl chains and/or the removal of phosphate groups lead to a strong decrease in biological activities.^5^ Although, the relationship between lipid-A chemical structure and immune response is well established, much less is known about the physical state of the biologically active lipid-A aggregates. While LBP recognizes and binds lipid-A aggregates, only its monomeric form is transferred by LBP to the CD14 receptor (at low LPS concentrations) or to serum lipoproteins (at high LPS concentrations).^6−8^ Lipid-A can form different aggregation states in water [*e.g.,* micellar (M), lamellar (L), hexagonal (H_I_), inverted hexagonal (H_II_), and nonlamellar cubic (Q)], often with the co-existence of two states (*e.g.,* Q and M).^9−11^ It has been proposed that the high endotoxic activity of lipid-A is associated to conical conformations forming cubic inverted aggregates, whereas cylindrical lipid-A conformations favor lamellar aggregates with low or no endotoxic activity.^9,12−14^

The accurate modeling of the chemical complexity of glycolipids across different scales is an extraordinary challenge.^15−17^ The carbohydrate region of LPS aggregates has numerous charged phosphate groups neutralized by an equally large number of cations. It was shown by SANS measurements^18,19^ and atomistic simulations^20,21^ that the carbohydrate region is fully hydrated with water molecules penetrating the lipid-A hydrophobic region deeper than observed for phospholipid membranes. In the last decade, there has been continuous efforts by several groups to develop atomistic^20−26^ and CG models^27−30^ that enables the adequate treatment of the unusually high charge density and hydration in LPS bilayers. Despite important progress on the computational and experimental fronts, the aggregation mechanism and structural dynamics of nonlamellar aggregates remains less explored and understood.

**Figure 1:**
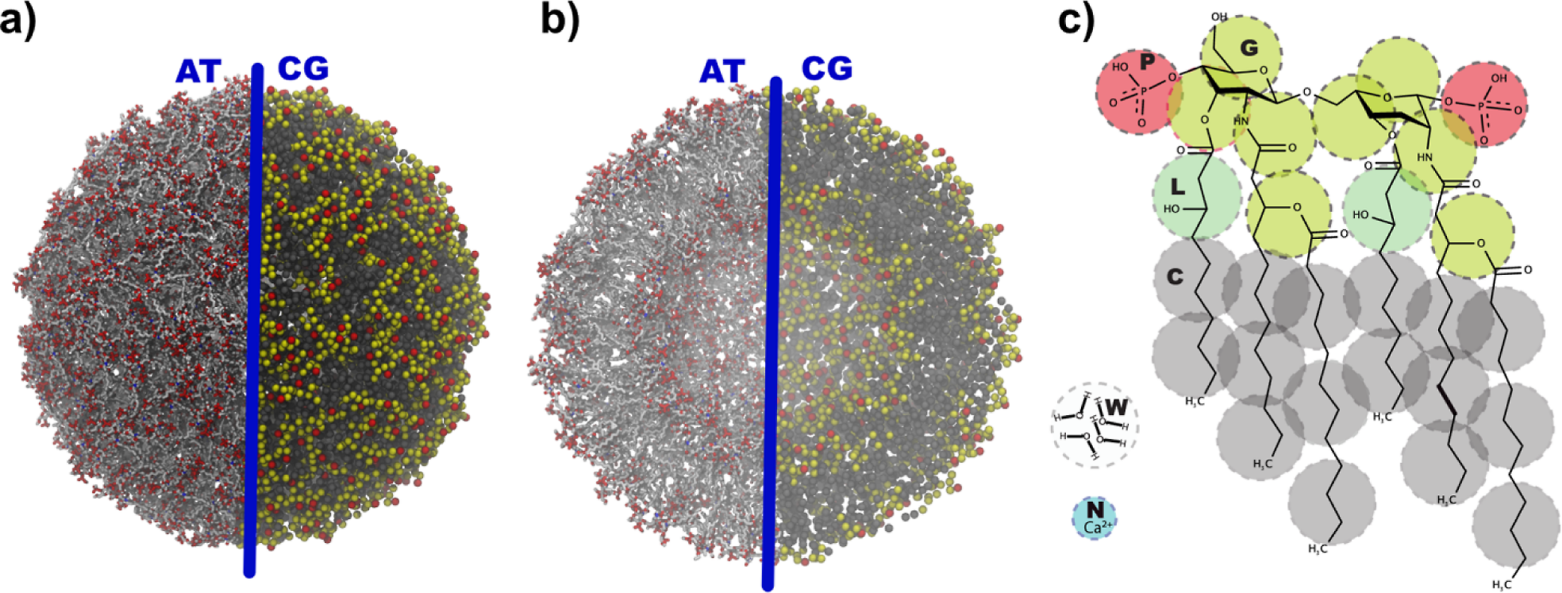
(a) Back-mapped atomistic (AT) configuration of the lipid-A vesicle together with the coarse-grained (CG) counterpart. (b) Cross section view of both AT and CG configuration of the lipid-A vesicle. Cations and water molecules are not shown for clarity. (c) Schematic overlay of the chemical structure and CG beads used in the hPF simulations.^30^ Bead types are indicated by the letters P, G, L, C, W, N, and the color scheme. Water bead contains ∼4H_2_O molecules.

We have previously investigated the behavior of lipid-A aggregates in solution by combining coarse-grained (CG) models of chemical variants of lipid-A (tetra- and hexa-acylated) with the hybrid particle-field (hPF)-molecular dynamics (MD) (hPF-MD) approach.^30^ The hPF-MD approach combines a microscopic molecular representation to density-based potential so that the explicit treatment of nonbonded pair interactions between particles is replaced by the evaluation of an external potential based on the local particle density.^31−33^ It also relies on a mean field treatment of short-range electrostatic interactions together with an implementation of Ewald summation for the treatment of long-range electrostatics.^34^ The hPF-MD simulations of random distributions of lipid A molecules and counterions in water^30^ reproduced the morphologies of the lamellar^18^ and nonlamellar phases,^9−11^ including the self-assembly of vesicle formation in aqueous solution. Most importantly, it provided a model mechanism for the spontaneous micellar aggregation and vesicle formation of this glycolipid.^30^ Hence, at low concentrations, lipid-A aggregated into small micelles that progressively fused into a single micelle composed of the total number of lipids in the simulation. At high concentrations, lipid-A aggregates evolved through the fusion of micelles into a vesicle which was stable at the μs timescale. In this work, we report for the first time the all-atom simulation of a lipid-A vesicle rebuilt *via* a back-mapping procedure from the CG vesicle identified in our previous report on lipid-A aggregation (Figure 1).

## Computational Methods

### Hybrid Particle-Field Approach

In the following, we report a brief introduction to hPF approach, while full description of the method and its extension to electrostatic interactions are reported in refs.^32−36^ In the hPF approach, a molecule is considered to interact with surrounding molecules through an external field. The field is built from the nonhomogeneous spatial density distributions of segments of independent molecules. The derivation of the external potential *V*_K_(***r***_***i***_) starting from the partition function is given in ref (32). It can be demonstrated that the density-dependent external potential can be written as
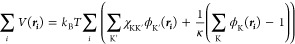
1where each component of the system is identified by the *K* index. The term  is the mean field interaction parameter between a *K*-type particle with the density field of particles of type *K*′. The term ϕ_K_(***r***_***i***_) is the density field of the species *K* at position ***r***, and κ is the compressibility term. *T* is the temperature of the system and *k*_B_ is the Boltzmann constant. Considering a simple case, a system composed of two types A and B, the mean field potential acting on a single particle A at position ***r*** is
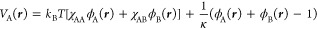
2

Then, the force acting particle A at position ***r*** is
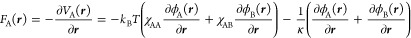
3

### Electrostatic Interaction Treatment in the hPF Approach

The electrostatic interactions between charged particles are evaluated through an electric field (EF) depending on the spatially inhomogeneous distributions of charge densities.^35^ EF can be represented by dividing the simulation box (***L***_1_,***L***_2_,***L***_3_) into *N*_1_ × *N*_2_ × *N*_3_ cells (the *N*_α_ = number of cells in the direction ***L***_α_ for α = 1,2,3). The location of lattice points is given by *l* = *l*_1_***L***_1_/*N*_1_,*l*_2_***L***_2_/*N*_2_,*l*_3_***L***_3_/*N*_3_, where *l*_α_ is an integer number 0 ≤ *l*_α_ < *N*_α_ The total Coulomb energy can be written as
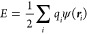
4where *q* is the reduced charge and ψ(***r***) is the electrostatic potential. Collecting the contribution over *i*-th particles gives us the ψ(***r***)
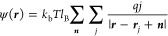
5The outer sum over ***n*** goes, with periodic boundary conditions, over the vectors ***n*** – *n*_1_***L***_1_ + *n*_2_***L***_2_ + *n*_3_***L***_3_. The Bjerrum length is , where *e* is the elementary charge and ϵ_0_,ϵ_r_ are the vacuum permittivity and the relative dielectric constant of the medium, respectively. The ψ(***r***) can be separated in long and short range by using the Ewald summation
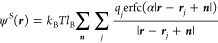
6
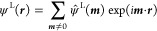
7The term  on the right hand of eq 7 is the long-range contribution of the electrostatic potential in the reciprocal space. Considering the Gaussian distribution of charge density, it is possible to solve the Poisson’s equation in the reciprocal space and to get 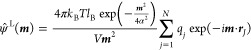
8where *V* is the box volume and ***m*** = 2π(*m*_1_***L***_1_^*^ + *m*_2_***L***_2_^*^ + *m*_1_***L***_1_^*^). ***L***_α_^*^ are the conjugated reciprocal vectors defined by the relations ***L***_α_^*^·***L***_β_ = δ_αβ_;α,β = 1,2,3.

The long-range contribution of the electrostatic potential at a lattice point of special position (***l***) can be written by using the discrete Fourier transform (DFT) in the following way
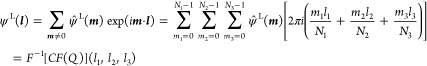
9where *Q* is the charge density at lattice points and *F*(*Q*) is the DFT. *F*^–1^ is the inverse DFT. Because of the hPF approach only mean field parameters are applicable, the short-range electrostatic interactions which are usually considered as pairwise interactions in standard MD can be evaluated in the following way. Using the Flory–Huggins approach for the lattice model, it is possible to evaluate the χ_e_ parameter for the short-range part of electrostatic interactions
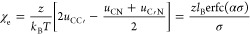
10where *z* is the coordination number (*z* = 6 for a 3D cubic lattice) while , *u*_CN_, and  are the pairwise short-range electrostatic energies between a pair of adjacent lattice sites (. σ is related to the diameter of particles. The term *u*_CN_ =  is for lattice sites occupied by one particle with (*e*) and other one being neutral. The short-range part of the electrostatic potential ψ^S^(***l***) can be obtained in the density field way^32^
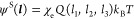
11

### hPF CG Lipid-A Model

In this study, we adopt the CG representation of the hPF model of lipid A developed in our previous work.^30^ The molecular model employed a CG mapping of four heavy atoms per bead and explicit electrostatic interactions to accurately represent divalent counterions (Figure 1c). The hPF simulations using the optimized parameter set successfully reproduced the lamellar phase of the reference all-atom models for lipid-A membranes with good qualitative and quantitative agreement to several structural properties.^30^ The functional form of bonded and nonbonded interacting potentials and parameters are presented in the Supporting Information.

### Back-Mapping Procedure to Build the Atomistic Lipid-A Vesicle

**Figure 2:**
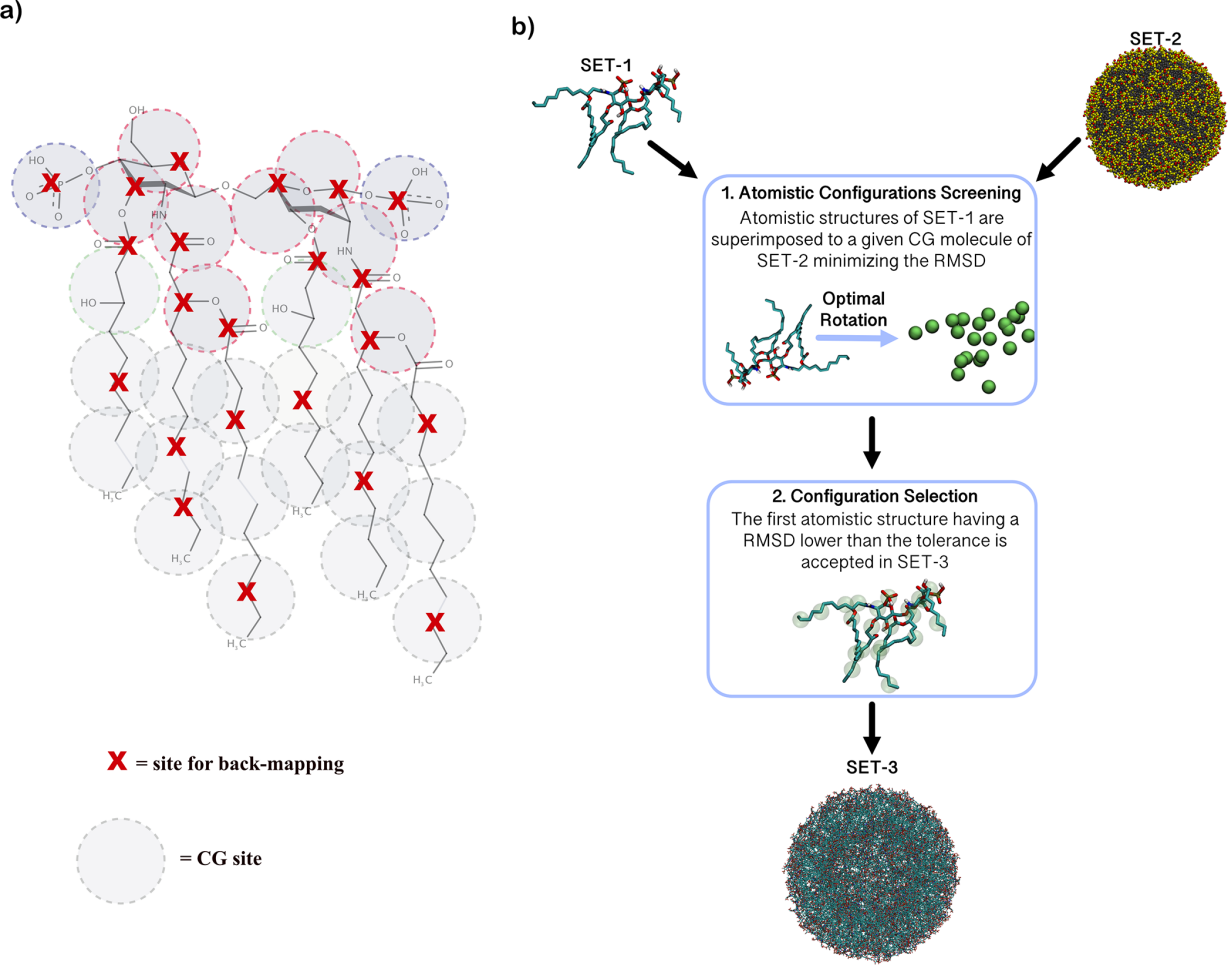
(a) Back-mapping sites, labeled with a red cross, used for the reconstruction of the all-atom vesicle. (b) Back-mapping scheme. SET 1 corresponds to atomistic configurations obtained from the lipid A atomistic trajectories. SET 2 is the given CG configuration obtained from hPF simulation to be back-mapped. SET 3 is the back-mapped atomistic configuration.

The CG equilibrium configuration of the lipid A vesicle used as a template to build the equivalent all-atom configuration has been taken from our previous work.^30^ The equilibrium CG configuration of the vesicle has been obtained *via* self-assembly from the hPF simulation. In the current work, the adopted back-mapping procedure is based on rigid superposition (rotation) of lipid A atomistic models on the CG ones obtained from the hPF simulations.^30^ A simplified sketch of such a procedure is shown in Figure 2. For a given CG molecule of lipid-A, several trial atomistic structures, belonging to a structure library, are superimposed to minimize the root mean square deviation (rmsd) between the center of CG beads and the corresponding atomistic sites. The quality of the final rebuilt structure is a function of the richness of the library, in terms of different atomistic configurations available to match the equivalent CG ones. To have a large collection of atomistic configurations, the structure library is made of a configuration taken from an atomistic MD simulation of a lipid-A bilayer in the fluid state. For this study, an atomistic simulation composed of 256 lipid-A molecules distributed in an 8 × 8 arrangement layer, simulated at 300 K for 500 ns, has been utilized. From this simulation, 5000 configurations have been used. For a lipid A molecule, after a rotation is able to maximize the superposition between the center of the CG beads and their corresponding atomic sites (Figure 2a), a given trial atomistic structure is accepted if the maximum value obtained for the rmsd between two sites is smaller than a fixed tolerance. For the system under investigation, a value of tolerance of 0.2 nm has been found to be a reasonable choice.

### Setup and Simulation of the Atomistic Models

The initial all-atom configuration of the lipid-A vesicle was reconstructed *via* a back-mapping procedure.^37,38^ An equilibrium configuration of the hexa-acylated CG lipid-A vesicle was selected from the hPF CG simulation of the lipid-A vesicle^30^ (Figure 1) and backmapped *via* spatial superposition (rotation) onto the all-atom configuration. MD simulation was performed for a lipid vesicle composed of 644 hexa-acylated lipid-A molecules (440 in the outer leaflet and 204 in the inner leaflet). To neutralize a charge of −2 e per lipid-A unit, 204 and 440 calcium ions were added in the interior and exterior of the vesicle, respectively. The SPC water model was used as the system solvent.^39,40^ The GROMOS 45A4/53A6 functional form for carbohydrates^41^ and parameter extensions for glycolipids^25^ were used. The vesicle structure was submitted to a multi-stage energy minimization process. The first stage in the process dealt with the spatial accommodation of the lipids to maximize the interaction between each molecule. To achieve that, 2,500,000 steps of the steepest descent algorithm were applied with an initial step-size of 0.001 nm and a tolerance of 0.1 kJ/mol. During this first stage, the lipid charges were removed in such a way that the forces observed between different molecules were only derived from the van der Waals potential. In the second stage, charges were added to the lipids, as well as calcium ions in sufficient quantity to neutralize the total charge of the system. The system geometry was optimized through 2,500,000 steps of the steepest descent algorithm with an initial step-size of 0.001 nm and a tolerance of 0.1 kJ/mol. The system was subsequently solvated with 204,592 molecules of water outside the vesicle and 5707 molecules inside the vesicle cavity, totalizing 210,299 solvent molecules. The full system was again geometry-optimized with the steepest descent algorithm. The system was slowly thermalized through five simulations in the *NVT* ensemble, at 100, 150, 200, 250, and 300 K, each with 5,000,000 steps.^42^ The temperature was kept constant by applying a velocity-rescaling thermostat with a coupling constant of 0.4 ps.^43^ Each of the equilibration steps was performed using the leapfrog integrator.^44^ The integration step used was 1 fs. The geometry of the water molecules and the size of the bonds between the atoms composing the solute were constrained to remain constant throughout the simulation using the LINCS algorithm.^45^ A cutoff radius of 1.2 nm was used to optimize the calculation of electrostatic and van der Waals interactions. Beyond this distance, the electrostatic interactions were treated through the particle mesh Ewald approximation.^46^ The positions of the atoms, as well as the potential and kinetic energies, and the velocities of each atom in the system were stored every 1000 simulation steps. After finishing the thermalization step in the *NVT* ensemble, an equilibration step followed for 500 ns, at 300 K, preceding the data production step. In this last step, a simulation was performed in the *NPT* ensemble for 300 ns, applying the same parameters defined for the previous simulations but now adopting the Berendsen barostat to control the pressure throughout the simulation.^47^ The pressure coupling for this simulation followed an isotropic scheme, with a coupling constant of 0.4 ps, a reference value of 1.0 bar, and the isothermal compressibility was defined as 4.5 × 10^–5^ bar^–1^, corresponding to the appropriate value for water.^20^ Finally, the production step of the MD simulation of the vesicle was performed with an integration step of 2 fs and for 300 ns. The simulation was carried out with GROMACS v5.0.7.^48^ Analyses of the trajectories were performed with GROMACS and SuAVE.^49,50^

## Results and Discussion

### Curvature Influences Physical Properties of the Lipid-A Aggregates

**Figure 3:**
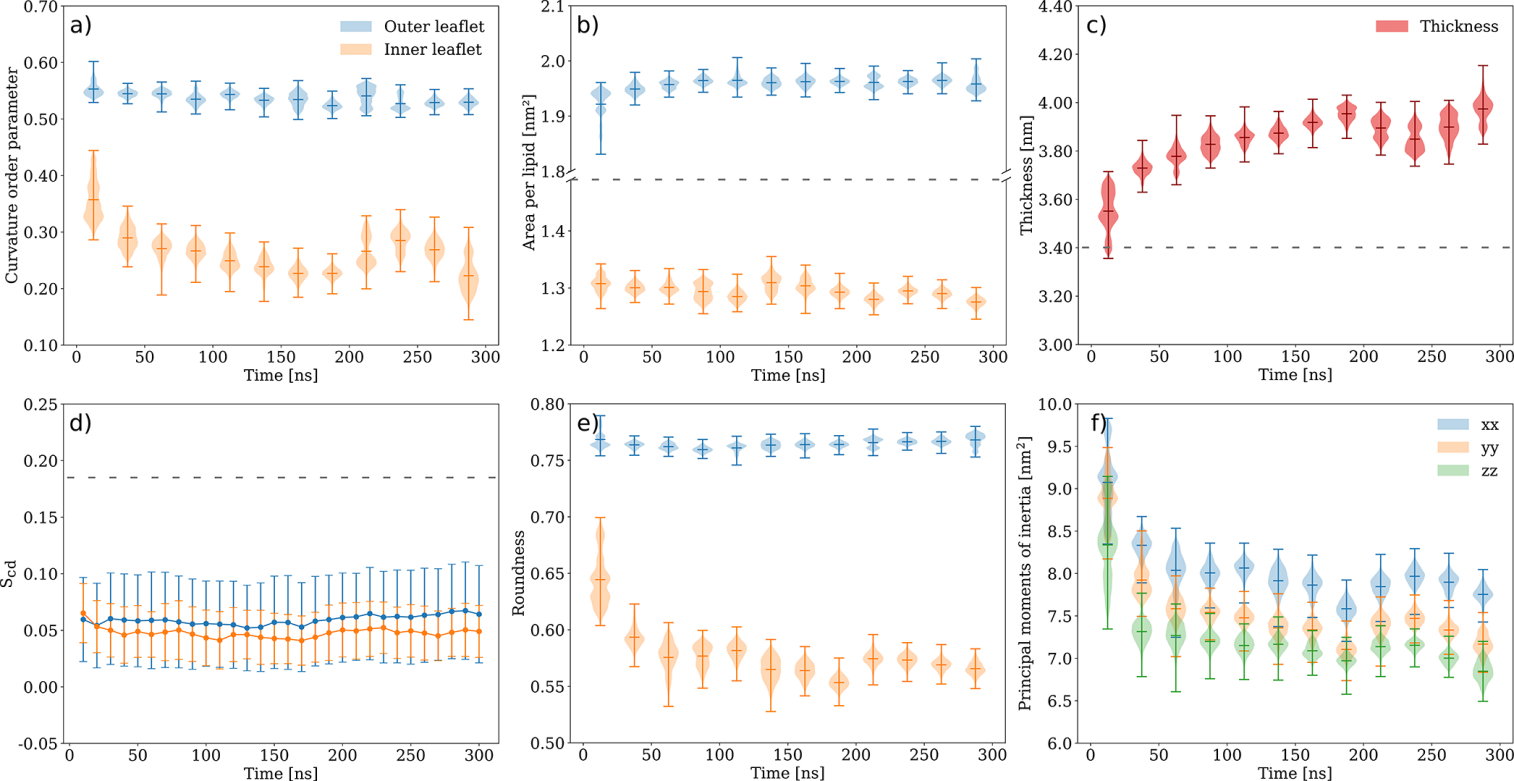
Time-dependent structural properties. (a) Curvature order parameter. (b) Area per lipid (*A*_L_). (c) Bilayer thickness. (d) Carbon-deuterium orientational order parameter (*S*_CD_). (e) Roundness index. (f) Principal moments of inertia. Data for the outer and inner leaflets are shown in blue and orange, respectively. Average property values for the lamellar arrangement^25^ of the hexa-acylated lipid-A are shown as dashed lines. Averages were run over time windows of 25 ns with the minimum, maximum, and average values shown for the respective time intervals.

The vesicle geometry imposes spatial restrains on the conformation and packing of the hexa-acylated lipid-A aggregates resulting in physical properties that differ from those in the corresponding lamellar arrangement and simulated under identical conditions (Figure 3). Furthermore, as curvature also differs between the two leaflets of the vesicle (Figure 3a), so do the physical properties extracted from the inner and outer leaflets (Figure 3b–f). The effect of curvature on the molecular and supramolecular dynamics of lipid-A can be assessed through the comparison of structural quantities from simulations of the vesicle and lamellar membrane under identical conditions (Figure 3). The average area per lipid (*A*_L_) in the vesicle outer and inner leaflets was 1.9 and 1.3 nm^2^, respectively (Figure 3b). For comparison, the lamellar arrangement had an average *A*_L_ of 1.8 nm^2^.^25^ On the average, the vesicle membrane thickness (*D*_HH_) was 0.4 nm larger than the lamellar arrangement whereas the carbon-deuterium order parameters (*S*_CD_) were nearly 4 times lower in the vesicle than in the bilayer (Figure 3c,d). Therefore, the vesicle has less ordered acyl chains when compared to the lamellar arrangement.

The vesicle morphology dynamics can be probed *via* the time evolution of the roundness index and principal moments of inertia (Figure 3e,f). The roundness of the initial configuration derived from the hPF-MD simulations gradually evolved to a less spherical shape in the atomistic simulations (Figure 3e,f). The decrease of the vesicle sphericity is also consistent with the decrease of the curvature order parameter (Figure 3a), particularly noticeable for the inner leaflet (Figure 3e). The magnitude of the vesicle shape deviation from a perfect sphere is proportional to the inequality of inertia moments. Hence, an oblate or discoid morphology can be excluded as each moment of inertia takes on a different value (Figure 3f), and the shape of vesicle can be regarded, on average, as quasi-spherical.

**Figure 4:**
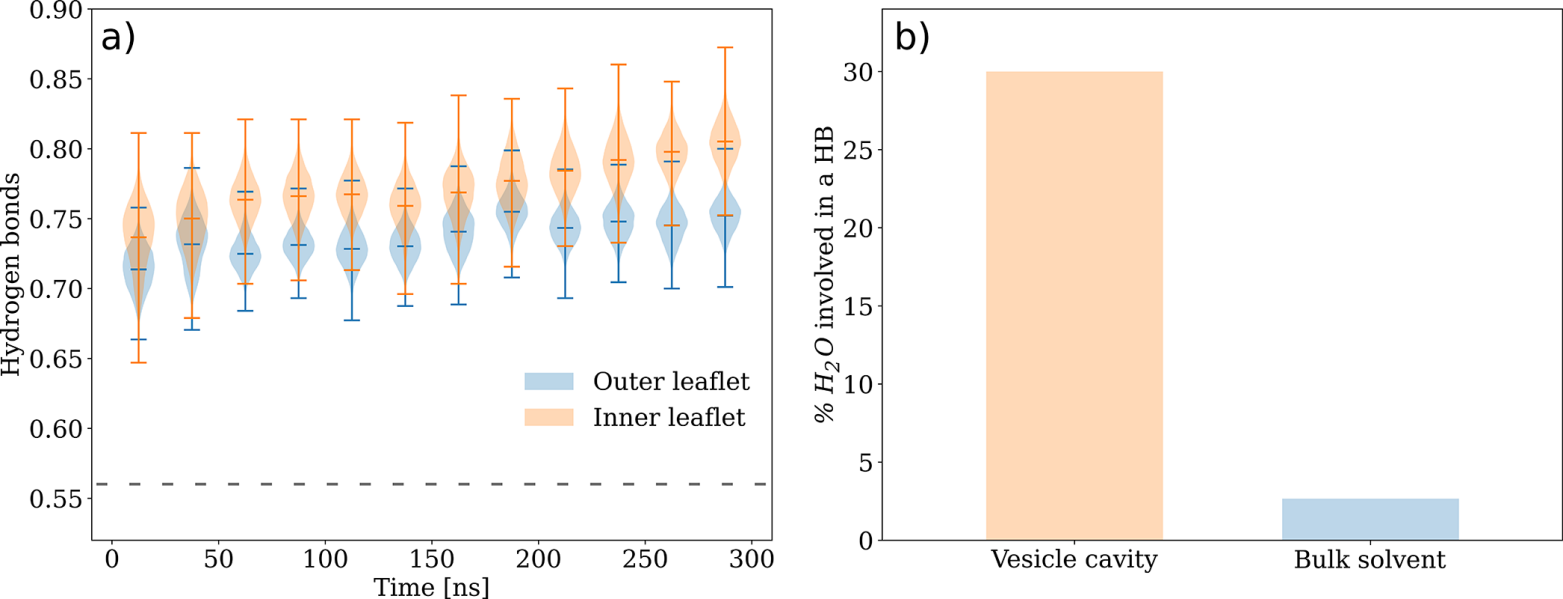
Time-averaged hydrogen bonds between phosphate groups and polar groups in the lipid-A molecule. (a) Number of hydrogen bonds per phosphate group. Dashed line represents the average number of hydrogen bonds in the lamellar arrangement of hexa-acylated lipid-A. (b) Percentage of water molecules in the vesicle cavity and the bulk solvent involved in at least one hydrogen bond interaction with phosphate groups in lipid-A. Hydrogen bonds were calculated using a cutoff distance of 0.35 nm and an angle of 30° between X-D---A (D = donor, A = acceptor, X = atom bonded to the donor).

The local interactions between lipid-A molecules and surroundings (water and counterions) play an important role on the variation of the vesicle shape. It has been experimentally shown that LPS aggregation requires specific divalent cations, commonly magnesium and calcium which are the most abundant divalent cations in Gram-negative bacteria.^51−53^ In the simulation, Ca^2+^ counterions are placed in the vicinity of phosphate groups to neutralize the negative charges of phosphate groups and allow for lipid-A aggregation. The high concentration of counterions in the vesicle cavity leads to a higher coordination number (CN) of the phosphate group against Ca^2+^ counterions in the inner leaflet (0.586 ± 0.001) when compared to the outer leaflet (0.255 ± 0.001) (Table 1). This behavior is accompanied by a 6-fold increase in the number of hydrogen bonds between water molecules (donors) and the phosphates (acceptors) for lipid-A in the inner leaflet versus the outer leaflet (Figure 4b). The CNs for atom pairs PO_4_/Ca^2+^, PO_4_/OW(H_2_O), and Ca^2+^/OW(H_2_O) in the vesicle and lamellar arrangements are presented in Table 1. It was previously shown that the hydration, ionic valence, and cross-linking propensities of cations determine the stability of LPS membranes.^54^ As result, it can tolerate higher hydration levels if cross-linked by divalent counterions. Ca^2+^ cations can effectively cross-link the phosphate groups from distinct lipid-A molecules increasing the molecular packing^55^ of the spatially constrained inner leaflet and stabilizing the vesicle supramolecular structure. It is also interesting to note that the lipid-A phosphate groups and Ca^2+^ cations have higher CNs with water molecules in the lamellar arrangement than in the outer leaflet (Table 1). This is expected to stabilize the hydrophobic core of the vesicle as its outer leaflet exhibits a less compact packing compared to the inner leaflet and the lamellar arrangement.

**Table 1:** Coordination Numbers of Major Functional Groups in the Inner and Outer Leaflet of the Lipid-A Vesicle and the Lamellar Arrangement (See Ref (25))

	PO_4_/Ca_2+_	PO_4_/OW(H_2_O)	Ca^2+^/OW(H_2_O)	*D* (water)
vesicle inner leaflet	0.586 (0.001)	6.49 (0.02)	5.654 (0.003)	2.4 (0.1)^a^
vesicle outer leaflet	0.255 (0.001)	6.96 (0.03)	6.992 (0.003)	3.5 (0.3)^b^
lamellar bilayer	0.104 (0.001)	7.45 (0.03)	7.640 (0.001)	2.7 (0.1)^b^

a Self-diffusion coefficient *D* [10^–5^ cm^2^/s] of water molecules in the vesicle cavity.

b The bulk solvent surrounding the lipid-A vesicle and the lipid-A bilayer. Averages were obtained over the last 100 ns of simulation.

### Water Permeation across the Lipid-A Vesicle

Neutron diffraction measurements have shown that water permeates the entire length of the LPS bilayer including the hydrophobic core and the terminal methyl groups in both gel (Lβ) and liquid crystalline (Lα) phases.^18^ Atomistic simulations of the bacterial OM model containing LPS (outer leaflet) and DPPE (inner leaflet) have also shown a deeper water penetration in the hydrophobic region in the LPS leaflet.^20^ Furthermore, the morphology of LPS aggregates and its corresponding transition temperatures are greatly influenced by the level of hydration. Fully hydrated suspensions of LPS can adopt a variety of nonlamellar morphologies, and the formation of bilayers is highly dependent on the amount of water present in the hydrophobic core.^18^

**Figure 5:**
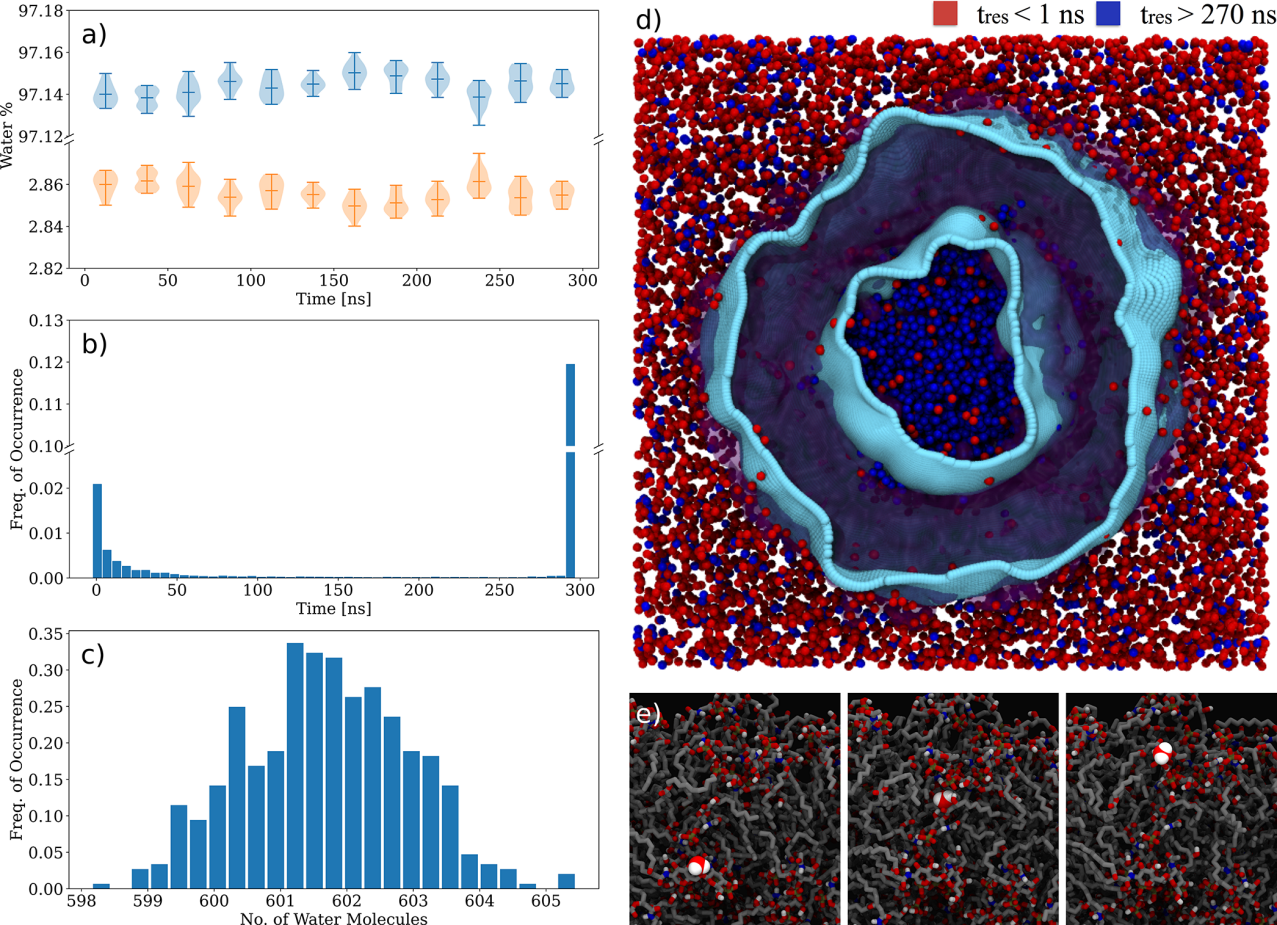
Water permeation across the lipid-A vesicle. (a) Time-dependent percentage of water molecules inside (orange) and outside (blue) of the vesicle. (b) Temporal frequency of water molecules crossing, at least once, the vesicle bilayer. (c) Distribution of number of water molecules crossing the vesicle bilayer. Each bar is averaged over 10 ns of the MD trajectory. (d) Representation of water molecules as function of residence times in the vesicle bilayer. Water molecules entering the vesicle for periods shorter than 1 ns (inclusive 0 ns) are depicted in red whereas water molecules remaining in the vesicle for longer than 270 ns are depicted in blue. (e) Snapshots of a selected water molecule crossing the vesicle bilayer through interactions with the phosphate and hydroxyl groups of the *N*-acetylglucosamine head groups. The vesicle bilayer surfaces are rendered in cyan and the hydrophobic region in between the surfaces are rendered as a transparent purple surface. Analyses were performed for 300 ns of simulation.

We investigated the dynamics of water permeation across the vesicle (Figure 5). Although the number of water molecules inside and outside the vesicle cavity does not vary extensively, there is a residual fraction of molecules permeating across the vesicle bilayer (Figure 5a). The time distributions calculated for each time a water molecule enters the membrane (Figure 5b,c) showed two regimes (Figure 5d). The set of water molecules with residence times of *t* > 270 ns were mostly confined inside the vesicle cavity (Figure 5d) and displayed lower diffusion coefficients (2.4 × 10^–5^ cm^2^/s) than in the bulk solvent (3.5 × 10^–5^ cm^2^/s) due to much larger number of hydrogen bond interactions (Figure 4b and Table 1).^56^ In contrast, the water molecules with residence times of *t* ≃ 1–50 ns underwent fast and reversible penetration of the membrane (Figure 5d). Most of these molecules belong to the bulk solvent outside the vesicle, but some of them (Figure 5c) could passively permeate the vesicle membrane inward and outward (Figure 5d). Lipid-A has numerous hydrogen bond donors/acceptors not only in the *N*-acetylglucosamine ring but also in the upper region of the acyl chains (Figure 1c). In our atomistic simulations, the water permeation appears to be facilitated by hydrogen bonds between membrane-crossing water molecules and these acceptor/donor groups in lipid-A (Figure 3e). Such a molecular mechanism may provide a rationale for the higher water permeation rates for LPS compared to phospholipid membranes^18,20^ and suggests that water permeation rates across OM vesicles can be modulated by chemical variations of the lipid-A structure.

## Conclusions

Lipid-A are MAMPs specifically identified by eukaryotic PRRs during Gram-negative bacterial infections. Therefore, the innate immune response is highly dependent on the chemical structure of lipid-A and the physical state of the biologically active lipid-A aggregates, but the latter is not well understood. Previous investigations of the aggregation behavior of lipid-A in solution *via* the hPF-MD approach provided a model mechanism for its spontaneous micellar aggregation and vesicle formation. In this work, we investigated the stability and physical properties of the atomistic simulation of the hPF-MD-generated lipid-A vesicle rebuilt *via* a back-mapping procedure from the CG model. It was found that the vesicle curvature significantly influences the conformation and packing of the lipid-A aggregates. The vesicle core is stabilized by the tight molecular packing of the inner leaflet and the electrostatic interactions between the phosphate and hydroxyl groups of the *N*-acetylglucosamine with the confined water molecules and ions in the vesicle cavity. The shape of the vesicle can be considered quasi-spherical for the simulated timescale. The outer leaflet has a lower molecular packing compared to both the inner leaflet and the lamellar arrangement of lipid-A. On average, *S*_CD_ values were nearly 4 times lower in the lipid-A vesicle than in the corresponding bilayer. The comparative decrease of the acyl chain order in the vesicle allows for the passive inward and outward permeation of a small fraction of water molecules. The water permeation may be facilitated by hydrogen bonds to the numerous hydrogen bond donors/acceptors and the flexible *N*-acetylglucosamine ring in lipid-A. These findings provide a rationale for the previously observed higher water permeation rates for LPS compared to phospholipid membranes and imply that water permeation rates across OM vesicles can be modulated by chemical variations of the lipid-A structure.

## Supporting Information Available

Parameters for the hPF CG lipid-A model, bond terms, angle terms, and interaction matrix (PDF)
