# Supplementary material for: Exploring the Molecular Dynamics of a Lipid-A Vesicle at the Atom Level: Morphology and Permeation Mechanism
Source: J Phys Chem B. 2023 Jul 19;127(30):6694–702. doi: 10.1021/acs.jpcb.3c02848 (PMC10405212; doi:10.1021/acs.jpcb.3c02848)
Supplement: Supplementary file 1 — jp3c02848_si_001.pdf [file jp3c02848_si_001.pdf]

Supporting Information

Exploring the Molecular Dynamics of a Lipid-A Vesicle at the Atom Level: Morphology and Permeation Mechanism

Denys E. S. Santos[1], Antonio de Nicola[2,3,], Vinicius Firmino[4],*

Giuseppe Milano[5], Thereza A. Soares[4,6]*

[1]Departmento de Química Fundamental, Universidade Federal de Pernambuco, 50740-560, Recife, Brazil

[2]Scuola Superiore Meridionale, Largo S. Marcellino 10, 80138 Napoli, Italy.

[3]Graduate School of Organic Materials Science, Yamagata University, Yonezawa, Yamagata, 992-8510, Japan

[4]Departamento de Química, Faculdade de Filosofia, Ciências e Letras de Ribeirão Preto, Universidade de São Paulo, 14040-901, Ribeirão Preto, Brazil

[5]Department of Chemical, Materials and Production Engineering, University of Naples Federico II, Piazzale Tecchio 80, 80125 Napoli, Italy

[6]Hylleraas Centre for Quantum Molecular Sciences, University of Oslo,

0315 Oslo, Norway

Corresponding Authors

a.denicola@ssmeridionale.it, thereza.soares@usp.br

Parameters for hPF Coarse-Grained (CG) Lipid-A Model.

The functional form of bonded and non-bonded interacting potentials and parameters for the hexaacylated Lipid-A are defined[1,2] as follow:

$$V_{bond} = \frac{K_{bond}}{2}(r_{ij} - r_0)^2 \quad \text{(S12)}$$

Table S1. Bond Terms

Bond Type	K_{bond} (kcal/mol/Ang.^{2})	r_0 (Ang.)
L-G	1250	0.47
L-C	1250	0.47
G-P	1250	0.47
G-G	1000	0.47
G-C	1250	0.47
C-C	1250	0.47

$$V_{angle} = \frac{K_{angle}}{2}(\theta_{ij} - \theta_0)^2 \qquad \text{(S13)}$$

Table S2. Angle Terms

Angle Type	K_{angle} (kcal/mol/rad^2)	θ_0 (deg.)
G-G-G	850	120
G-G-L	25	120
L-C-C	25	180
C-C-C	25	180

Table S3. Interaction matrix $\chi_{KK'} \times RT$ $(kJ\ mol^{-1})$.

$\chi_{KK'}$	**L**	**P**	**G**	**C**	**Ion (N)**	**Water (W)**
**L**	0	0	4.5	13.25	0	0
**P**	0	0	4.5	20	-7.2	-3.6
**G**	4.5	4.5	0	8.3	0	4.5
**C**	13.25	20	8.3	0	13.25	33.75
**Ion (N)**	0	-7.2	0	13.25	0	0
**Water (W)**	0	-3.6	4.5	33.75	0	0

References

(1) Milano, G.; Kawakatsu, T. Hybrid Particle-Field Molecular Dynamics Simulations for Dense Polymer Systems. *J. Chem. Phys.* **2009**, *130* (21), 214106. https://doi.org/10.1063/1.3142103.

(2) De Nicola, A.; Soares, T. A.; Santos, D. E. S.; Bore, S. L.; Sevink, G. J. A.; Cascella, M.; Milano, G. Aggregation of Lipid A Variants: A Hybrid Particle-Field Model. *Biochimica et Biophysica Acta (BBA) - General Subjects* **2020**, 129570. https://doi.org/10.1016/j.bbagen.2020.129570.
